# The multiple faces of extracellular vesicles released by microglia: Where are we 10 years after?

**DOI:** 10.3389/fncel.2022.984690

**Published:** 2022-09-13

**Authors:** Martina Gabrielli, Stefano Raffaele, Marta Fumagalli, Claudia Verderio

**Affiliations:** ^1^CNR Institute of Neuroscience, Vedano al Lambro, Italy; ^2^Department of Pharmacological and Biomolecular Sciences, Università degli Studi di Milano, Milan, Italy

**Keywords:** microglia, extracellular vesicles, heterogeneity, cell-to-cell communication, exosomes, microvesicles

## Abstract

As resident component of the innate immunity in the central nervous system (CNS), microglia are key players in pathology. However, they also exert fundamental roles in brain development and homeostasis maintenance. They are extremely sensitive and plastic, as they assiduously monitor the environment, adapting their function in response to stimuli. On consequence, microglia may be defined a heterogeneous community of cells in a dynamic equilibrium. Extracellular vesicles (EVs) released by microglia mirror the dynamic nature of their donor cells, exerting important and versatile functions in the CNS as unbounded conveyors of bioactive signals. In this review, we summarize the current knowledge on EVs released by microglia, highlighting their heterogeneous properties and multifaceted effects.

## Introduction

Microglia are commonly defined as the resident immune cells of the CNS. Once considered vicious players in pathology, these long-lived and self-maintained cells are now acknowledged for the versatility and complexity of their functions.

Microglia is a unique, peculiar cell type. Unlike neural cells, which have a neuroectodermal origin, microglia originate from early erythroid myeloid progenitors, belonging to the mononuclear phagocyte system (Ginhoux et al., 2010). Yet, microglia are divergent from peripheral tissue phagocytes, displaying distinct transcriptomes and epigenomes (Gosselin et al., 2014; Lavin et al., 2014).

### The multiplicity of microglia activation states

It is typical of microglia to sense and react to the smallest stimulus, finely shaping themselves in response. Depending on the signals they receive from the environment, microglia can acquire a variety of different activation states, which are difficult to discriminate *in vivo* on the base of phenotypic markers.

An outdated paradigm described three main distinct microglial states: M0, M1, and M2 (Martinez and Gordon, 2014; Ransohoff, 2016). M0 was for the so-called resting microglia, ideally found in the healthy brain. M1 and M2 represented two opposite activation states: pro-inflammatory (initiating/contributing to injury) and pro-regenerative (devoted to the resolution of inflammation and tissue repair). M1 and M2 states can be induced *in vitro* by exposure to specific pro-inflammatory or anti-inflammatory cytokines. However, M1/M2 polarization is now recognized as a highly questionable and overly stereotyped model, very far from recapitulating *in vivo* microglia activation states (Ransohoff, 2016; Colonna and Butovsky, 2017).

Current understanding accounts for microglia in the healthy brain as homeostatic. Far from being inactive, homeostatic microglia tirelessly extend and retract their processes to sense every change in the environment (Davalos et al., 2005; Nimmerjahn et al., 2005). In physiological conditions, they monitor and modulate neuronal function, and sculpt neuronal networks during development (Salter and Stevens, 2017). They also support other CNS cells, promoting myelinogenesis and angiogenesis (Prinz et al., 2019).

In response to insults, microglia adapt their function to restore brain homeostasis (Sharma et al., 2020). Intriguingly, microglia response is influenced not only by the actual state of brain tissue but also by its previous “history,” revealing that microglia are subjected to immune memory. In fact, exposure to a peripherally applied inflammatory stimulus induces a long-lasting epigenetic reprogramming of microglia, modulating their responses to subsequent insults (Wendeln et al., 2018).

Under sustained activation, microglia undergo phenotypic specification into neurodegenerative or disease-associated microglia (MGnd/DAM) (Keren-Shaul et al., 2017; Krasemann et al., 2017; Mathys et al., 2017), whose role is pivotal in disease progression. DAM response arises as a protective mechanism to hamper neuronal damage upon broken homeostasis, but eventually evolves in a determinant of disease (Deczkowska et al., 2018). Single cell/nucleus RNA-seq analyses have distinguished multiple states within DAM populations (Keren-Shaul et al., 2017), including a DAM subcluster displaying a senescence-associated profile (Hu et al., 2021), suggesting a possible transition from early protective DAM to late stage toxic microglia, which lose their homeostatic and pro-resolving functions rather than acquire excessive inflammatory activities.

Once considered a secondary event, microglia primary involvement in pathology has been proven by genome-wide studies, showing that many risk genes for CNS disorders are expressed by microglia (Prinz et al., 2019; Bellenguez et al., 2022). Among the microglial genes that are associated with an increased risk for neurodegenerative diseases are progranulin and the triggering receptor expressed on myeloid cells 2 (TREM2). TREM2 regulates key genes involved in the switch from homeostatic to DAM state. Its loss of function locks microglia in a homeostatic state (Krasemann et al., 2017), while lack of progranulin causes an exaggerated microglial inflammation. Although loss of TREM2 and progranulin results in opposite activation states, both induce neurodegeneration, indicating that even locking microglia in homeostatic state may be detrimental (Gotzl et al., 2019), and pointing to the importance to balance microglial activity.

### Microglia heterogeneity

Microglia are highly heterogeneous cells and their distribution varies across brain regions (Lawson et al., 1990). They display distinct morphology depending on whether they are close to neuron cell bodies, dendrites, myelinated or not-myelinated axons, or vasculature. Their gene-expression profiles, self-renewal and turnover rates are different in distinct brain regions, reflecting the influence of local microenvironment (de Haas et al., 2008; Doorn et al., 2015; Grabert et al., 2016; De Biase et al., 2017; Gosselin et al., 2017; Ayata et al., 2018; Masuda et al., 2019). Various subclasses of microglia were identified in the CNS at distinct developmental stages (Matcovitch-Natan et al., 2016; Hammond et al., 2019; Li et al., 2019; Masuda et al., 2019), revealing that these cells display temporal diversity. Finally, divergence in microglia abundance, morphology, phagocytic capacity and gene expression were reported between sexes (Schwarz et al., 2012; Crain et al., 2013; Pimentel-Coelho et al., 2013; Butovsky et al., 2015; Dorfman et al., 2017; Hanamsagar et al., 2017; Krasemann et al., 2017; Villa et al., 2018; Weinhard et al., 2018b).

Hence, microglia are a community of cells diverse in their properties and functions, which co-exist at steady state and react differently to stimuli, always being in a dynamic equilibrium (Torres-Platas et al., 2014; Keren-Shaul et al., 2017; Krasemann et al., 2017; Stratoulias et al., 2019; Uriarte Huarte et al., 2021). Microglia heterogeneity depends both on *intrinsic* properties and functional specializations acquired by the cells during their maturation in the CNS (Stratoulias et al., 2019).

Although most studies have been performed in rodents, recent data indicate that microglia heterogeneity is also relevant to humans (Bottcher et al., 2019; Masuda et al., 2019). Eggen’s group documented extensive overlap of expression profiles between human and mouse microglia, which share the majority of genes and functions (Galatro et al., 2017). Murine and human microglia share some similarities also in disease. The expression profile of phagocytic/activated microglia in an amyloid mouse model is similar to that of human microglia associated to amyloid plaque in Alzheimer’s disease (AD) tissue (Gerrits et al., 2021). However, rodents cannot fully recapitulate human microglia genetics. For example, differences in the expression of genes changing during aging (less than 1 percent overlap of aging-associated genes between the species) or related to immune function (higher in humans) (Galatro et al., 2017) have been reported between human and murine microglia, and over half of the proteins associated with gene loci implicated in AD display less than 70% homology between the species (Hasselmann and Blurton-Jones, 2020). These species-specific differences highlight limitations to the relevance of data generated in mice for understanding the biology of human microglia.

### Extracellular vesicles released by microglia

Microglia interact with other cells in the CNS via several mechanisms, including cell-to-cell contact, secreted molecules, nanotubes (Scheiblich et al., 2021) and extracellular vesicle (EV) release.

EVs are lipid-encased nanoparticles conveying bioactive signals from donor to target cells. Carrying proteins, lipids and nucleic acids (DNA, RNA), EVs can release soluble factors in the extracellular space, present surface molecules to target cells or deliver their content to recipient cells upon endocytosis, phagocytosis or complete/partial fusion.

The most described EV subtypes have two distinct origins. So-called microvesicles stem directly from the plasma membrane, while exosomes are generated inside endosomes/multivesicular bodies and are released after multivesicular bodies fusion with the cell membrane. Given the impossibility to efficiently separate microvesicles from exosomes, EVs are currently distinguished based on their size, with large EVs (also called medium-large) being > 200 nm and small EVs < 100–200 nm, their biochemical composition and cell of origin, rather than the mechanism of biogenesis (Thery et al., 2018).

EV composition and function mirrors those of donor cells. Thus, microglia multiplicity and variety of activation modalities are inevitably reflected by EV heterogeneity in content and function. Given the extreme reactivity of these cells, not to be underestimated is the repercussion of experimental settings (i.e., different isolation systems/culture media/developmental stages, isolation from brain/body fluids/cultures) on microglia and their EV composition and action.

Regulated by environmental stimuli is also EV release. The classic trigger is ATP (Asai et al., 2015; Takenouchi et al., 2015; Drago et al., 2017; Lombardi et al., 2021). Relatively to large EVs, it is known that ATP activates the purinergic P2×7 receptor on the surface of microglia and evokes EV production in a process involving p38 MAP kinase and acid sphingomyelinase (Bianco et al., 2005, 2009). Recently, Mcfd2, Sepp1, and Sdc1 genes have been identified as regulators of ATP-induced small EV secretion from mouse microglia in a functional genome-wide short hairpin RNA library screening (Ruan et al., 2022). Among other stimuli promoting EV secretion from cultured microglia there are: pro-inflammatory cytokines (Colombo et al., 2018; Prada et al., 2018), IL-4 (Colombo et al., 2018; Prada et al., 2018; Lombardi et al., 2019; Raffaele et al., 2021), lipopolysaccharide (LPS) (Yang et al., 2018), capsaicin (Marrone et al., 2017), serotonin (Glebov et al., 2015), Wnt3a (Hooper et al., 2012), α-synuclein (Chang et al., 2013; Guo et al., 2020), ethanol (Mukherjee et al., 2020), manganese (Sarkar et al., 2019), fine particulate matter PM_2_._5_ (Chen et al., 2020). Conversely, cocaine has been reported to significantly decrease EV release from BV2 cell lines (Kumar et al., 2021).

*In vivo* EV production from microglia (and macrophages) is altered in pathological conditions, including Multiple Sclerosis (MS), AD and traumatic brain injury (TBI) (Verderio et al., 2012; Agosta et al., 2014; Joshi et al., 2014; Kumar et al., 2017; Liu et al., 2017; Dalla Costa et al., 2021; Gelibter et al., 2021). In TBI, EV release from microglia is mediated by activation of P2×7R, likely in response to the drastic increase in extracellular ATP released by damaged cells (Liu et al., 2017). In a humanized AD mouse model, plaque-associated microglia that phagocyte plaque-associated tau, apoptotic neurons and synapses, hyper-secrete tau-carrying EVs (Clayton et al., 2021). The measurement of microglial EV levels in body fluid is emerging as a useful parameter in diagnosis and prognosis, to monitor disease progression as well as treatment efficacy.

In this review, we will deepen into the heterogeneous nature of EVs released by microglia. Different microglial models and EV isolation methods are described with the intention to acknowledge the potential bias of each experimental setting. The available data on the composition of microglial EVs have been also examined to get insights into their biological functions. Finally, we summarize current knowledge on the effects of microglial EVs on the surrounding environment, pointing out that EVs may exert divergent functions based also on the type of recipient cells.

## How to obtain and study microglial extracellular vesicles

So far, scientists have exploited different microglia models to produce and isolate microglial EVs and study their features and functions, as reported in Table 1. A summary of EV purification methods is outlined in the Box 1.

**TABLE 1:** Microglial models for extracellular vesicle production and isolation.

Specie	Origin	Stimulus	Purification/Detection	EV type	References
Human	CHME-5 cell line	ATP, INF-*γ*, IL-4	Differential centrifugation/ flow cytometry	Large EVs	Colombo et al., 2018
	HMC3 cell line	Cocaine	Differential centrifugation	Small EVs	Kumar et al., 2020
	iPSC-derived microglia-like cells	LPS	Precipitation kit	Small EVs	Mallach et al., 2021a,b
		Potassium chloride, LPS	Differential centrifugation	Small EVs	You et al., 2022
	Post-mortem brain tissue		Density gradient + CD11b immuno-affinity capture	Small EVs	Cohn et al., 2021
	CSF		Flow cytometry (IB4)	Large EVs Large EVs Large EVs Large EVs Large EVs	Verderio et al., 2012 Joshi et al., 2014 Agosta et al., 2014 Dalla Costa et al., 2021 Gelibter et al., 2021
			CD11b immuno-affinity capture/ Flow cytometry (CD11b)	Small EVs	Guo et al., 2020
	Plasma		Precipitation kit + IB4 immuno-affinity capture	Small EVs	Scaroni et al., 2022
Mouse	Primary cultures	Unstimulated	Differential centrifugation	Small EVs Small EVs Small EVs	Peng et al., 2021 Potolicchio et al., 2005 Vinuesa et al., 2019
				Large/small EVs	Lecuyer et al., 2021
			Precipitation kit	Small EVs	Luong and Olson, 2021
			Density gradient	Small EVs	Potolicchio et al., 2005
		ATP	Differential centrifugation	Large EVs Total EVs Large EVs Small EVs	Marrone et al., 2017 Raffaele et al., 2021 Gabrielli et al., 2022 Serpe et al., 2021
		LPS + ATP	Differential centrifugation	Small EVs Small EVs	Asai et al., 2015 Serpe et al., 2021
			Size exclusion chromatography	Small EVs	Crotti et al., 2019
		Capsaicin	Differential centrifugation	Large EVs	Marrone et al., 2017
		Serotonin	Differential centrifugation		Glebov et al., 2015
		α-synuclein + ATP, LPS, cytokines	Precipitation kit	Small EVs Small EVs	Fan et al., 2019 Guo et al., 2020
		Oxygen-glucose deprivation	Precipitation kit + density gradient	Total EVs	Zhang et al., 2021
	Organotypic entorhino-hippocampal cultures	Ethanol	Differential centrifugation + flow cytometry (CD11b)	Large EVs	Coleman et al., 2017
	CD11b^+^ MACS isolated primary cultures	Unstimulated	Differential centrifugation	Large EVs	Kumar et al., 2017
		LPS + ATP	Differential centrifugation	Small EVs Small EVs	Ruan et al., 2020 Ruan et al., 2022
	BV2 cell line	Unstimulated	Differential centrifugation	Small EVs Total EVs Small EVs Small EVs Small EVs Small EVs	Huang S. et al., 2018 Casella et al., 2018 Vinuesa et al., 2019 Zhang et al., 2020 Van den Broek et al., 2020 Huang et al., 2022
			Differential centrifugation + precipitation kit	Small EVs	Ge et al., 2020
			Differential centrifugation + Gravity filtration	Large/small EVs /apoptotic bodies	Osteikoetxea et al., 2015
		ATP	Differential centrifugation	Large/small EVs Small EVs Large EVs	Grimaldi et al., 2019 Ruan et al., 2022 Gouwens et al., 2018
		Unstimulated vs. LPS	Differential centrifugation	Small EVs Large EVs Small EVs	Yang et al., 2018 Kumar et al., 2017 Fan et al., 2022
		LPS	Differential centrifugation/ precipitation kit	Small EVs Small EVs	Sarkar et al., 2019 Tian et al., 2019
		TNF	Differential centrifugation + size exclusion chromatography	Small EVs	Van den Broek et al., 2020
		ATP, INF-*γ*, IL-4	Differential centrifugation/ flow cytometry	Large EVs	Colombo et al., 2018
		IL-4	Differential centrifugation	Small EVs Small EVs Small EVs	Song et al., 2019 Li et al., 2021 Li et al., 2022
			Differential centrifugation + precipitation kit	Small EVs	Tian et al., 2019
			Density gradient	Small EVs	Liu et al., 2021
		Serotonin	Differential centrifugation	Small EVs	Glebov et al., 2015
		α-synuclein	Differential centrifugation	Small EVs	Chang et al., 2013 Xia et al., 2019
		Cocaine	Differential centrifugation	Small EVs	Coleman et al., 2017
		Ethanol	Differential centrifugation	Large EVs	Coleman et al., 2017
		PM_2_._5_	Differential centrifugation	Small EVs	Chen et al., 2020
		Enhanced hydrostatic pressure	Differential centrifugation	Small EVs	Aires et al., 2020
		Manganese	Differential centrifugation	Small EVs	Sarkar et al., 2019
	N9 cell line	Unstimulated	Differential centrifugation/ Density gradient	Small EVs	Potolicchio et al., 2005
		ATP	Differential centrifugation	Large/small EVs Large EVs	Bianco et al., 2009 Antonucci et al., 2012
		LPS + ATP	Annexin-V immuno-affinity capture	Large EVs	Bianco et al., 2005
	MG6 cell line	LPS + ATP	Differential centrifugation + precipitation kit	Large/small EVs	Takenouchi et al., 2015
	Brain tissue		Differential centrifugation + precipitation kit + immune-affinity capture (CD11b)	Small EVs Small EVs	Ge et al., 2020 Luong and Olson, 2021
	CSF		Differential centrifugation/ Flow cytometry (IB4)	Large EVs	Verderio et al., 2012
			Flow cytometry (IB4)	Large EVs	Colombo et al., 2018
	Blood		Differential centrifugation/ flow cytometry (P2Y12-CD45-AnnexinV)	Large EVs	Kumar et al., 2017
Rat	Primary cultures	Unstimulated vs. ATP	Differential centrifugation	Large/small EVs	Drago et al., 2017
		ATP	Differential centrifugation	Large EVs Large EVs Large EVs Large EVs Large EVs Large EVs Large/small EVs Total EVs	Verderio et al., 2012 Antonucci et al., 2012 Joshi et al., 2014 Gabrielli et al., 2015 Riganti et al., 2016 Prada et al., 2016 Prada et al., 2018 Lombardi et al., 2019
		LPS	Differential centrifugation	Small EVs	Cunha et al., 2016
		LPS + ATP	Annexin-V immuno-affinity capture	Large EVs Large EVs	Bianco et al., 2005 Verderio et al., 2012
		Wnt3a	Differential centrifugation	Small EVs	Hooper et al., 2012
		Ethanol	Differential centrifugation + precipitation kit	Small EVs	Mukherjee et al., 2020
	Retinal primary cultures	Elevated hydrostatic pressure	Differential centrifugation	Small EVs	Aires et al., 2020
	CD11b/c^+^ MACS isolated primary cultures	LPS	Differential centrifugation ± Size exclusion chromatography	Small EVs	Murgoci et al., 2020
	CSF		Differential centrifugation/ flow cytometry (IB4)	Large EVs	Verderio et al., 2012
Leech	Primary cultures	Unstimulated	Differential centrifugation ± density gradient	Small EVs Small EVs Small EVs	Raffo-Romero et al., 2018 Arab et al., 2019 Lemaire et al., 2019

**BOX 1 Extracellular vesicles isolation and purification methods.**

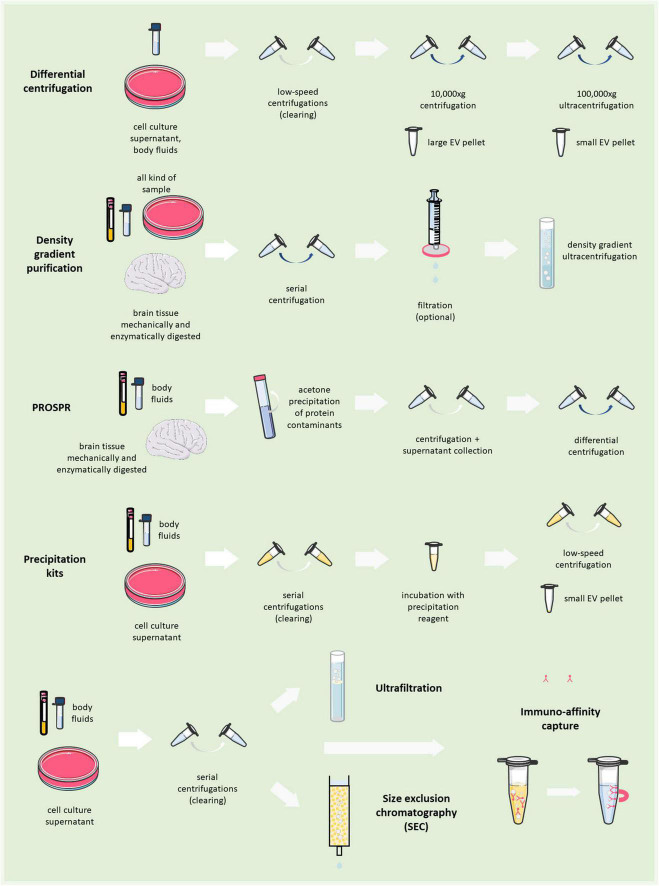

For many years differential centrifugation has been the gold standard to isolate total EVs from body fluids and culture media. It consists of a series of low-speed centrifugations, aimed at clearing the sample from dead cells, debris and larger particles, followed by a high-speed ultracentrifugation to collect pellets enriched in large EVs (>200 nm EVs; 10,000–20,000 × g × 30 min), or small EVs (<200 nm; 100,000–200,000 × g × 1–3 h). However, ultracentrifugation, especially at 100,000× *g*, may damage EVs and create artificial aggregates (Linares et al., 2015). In addition, by this method EVs are separated based on their dimension/density and not their subcellular origin. Co-isolation with protein aggregates or other contaminants is also possible. Therefore, to extract EVs from complex samples (e.g., tissues), differential centrifugation is followed by purification on density gradient made of sucrose (Perez-Gonzalez et al., 2012; Vella et al., 2017; Muraoka et al., 2020) or Optiprep iodixanol (Hurwitz et al., 2018; Crescitelli et al., 2021). Alternatively, to remove protein contaminants a clearance step by acetone precipitation can be exploited, which leaves EVs behind in suspension, allowing subsequent isolation of higher purity EVs by ultracentrifugation (PRotein Organic Solvent PRecipitation (PROSPR); Gallart-Palau et al., 2015, 2016, Mol Neurodegen). Precipitation of small EVs by Polyethylene Glycol (PEG) or other proprietary polymers from body fluids has caught on recently with the advance of EV isolation kits (Rider et al., 2016; Helwa et al., 2017), although these methods may also sediment contaminants along with EVs. Immunoaffinity capture allows the isolation of EVs using antibodies that recognize an EV marker conjugated with magnetic beads (or biotin-antibodies for recovery through streptavidin beads). After total EV isolation, microglial EVs can be isolated by immunoaffinity capture using antibodies recognizing myeloid/microglial markers (Scaroni et al., 2022; Cohn et al., 2021). Alternatively, size exclusion chromatography- or ultrafiltration-based methods are also employed. Parts of the figures were drawn by using pictures from Servier Medical Art. Servier Medical Art by Servier is licensed under a Creative Commons Attribution 3.0 Unported License (https://creativecommons.Org/licenses/by/3.0/).

Most studies used microglial EVs from *in vitro* rodent models, using both primary cultures [from C57BL/6 (Kumar et al., 2017; Raffaele et al., 2021; Gabrielli et al., 2022) or CD-1 mice (Asai et al., 2015; Ruan et al., 2020), Sprague-Dawley (Antonucci et al., 2012; Gabrielli et al., 2015; Riganti et al., 2016; Drago et al., 2017; Prada et al., 2018; Lombardi et al., 2019) or Wistar rats (Murgoci et al., 2020)] and microglia cell lines [murine BV2 (Hooper et al., 2012; Chang et al., 2013; Glebov et al., 2015; Kumar et al., 2017; Tian et al., 2019), N9 (Bianco et al., 2005; Potolicchio et al., 2005), MG6 (Takenouchi et al., 2015)]. Rodent primary cultures are usually established from day 0 to 3 newborns or late stage (18–21 gestational days) embryos as mixed glia cultures. Then, microglia are typically harvested from mixed cultures by shaking and maintained as pure cultures for a few (2–4) days only. Alternatively, microglia can be isolated from the rodent brain by magnetic activation cell sorting (MACS) (Krishnan et al., 2017; Kumar et al., 2017; Murgoci et al., 2020; Van den Broek et al., 2020; Ruan et al., 2022), a method that allows microglia isolation from adult brain and reduces the time of microglia maintenance in culture.

An interesting source for microglial EVs is microglia established from adult medicinal leech (*Hirudo medicinalis)* (Raffo-Romero et al., 2018). Leech microglia-derived EVs share several similarities to mammalian EVs, especially in their molecular composition (Raffo-Romero et al., 2018; Arab et al., 2019; Lemaire et al., 2019). Nevertheless, leech microglia contributed to rapid regeneration of the spinal cord and, therefore, the EVs they release may represent a source of regenerative factors lacking in mammalian microglia.

Compared to immortalized cell lines, primary microglia from rodents or leech are a source of EVs closer to the *in vivo* condition (Timmerman et al., 2018). Indeed, under defined culturing medium (added of TGF-β1 plus MCSF) primary mouse microglia can acquire a homeostatic-like signature, resembling freshly sorted adult cells (Butovsky et al., 2014). However, cell lines maintain the capability to respond to EV release stimuli such as ATP and LPS (Corradin et al., 1993; Takenouchi et al., 2005; Das et al., 2016) and are widely used due to their workability (e.g., ready to use, high proliferation rate, higher EV yield).

Human microglia have been only marginally employed to study EVs so far. Few studies employ EVs isolated from human microglia cell lines [HMC3 (Kumar et al., 2020); CHME-5 (Colombo et al., 2018)]. Other human cell lines (i.e., C20) display an altered response to LPS (Garcia-Mesa et al., 2017; Pozzo et al., 2019), while establishing primary microglia from post-mortem/cryo-preserved human brain tissue is still challenging. This limitation may be overcome by the analysis of EVs produced by microglia-like cells, differentiated from induced pluripotent stem cells (iPSC), as recently described (Mallach et al., 2021a,b; You et al., 2022). Several new protocols to generate human microglia *in vitro* from iPSCs have been reported, which try to mimic the cues that naturally drive microglia differentiation *in vivo*. However, studies comparing iPSC-derived microglia-like cells to *in vivo* reference microglia are still limited. Thus, it is still unclear whether microglia differentiated from iPSC cells fully recapitulate the human cells (Hasselmann and Blurton-Jones, 2020).

Importantly, EVs can not only be isolated *in vitro* from culture media, but also from body fluids (blood, CSF, saliva, tears, urine, etc …) or tissues, which contain EVs produced by many cell types. Thus, in principle, microglial EVs can be isolated from complex fluids or brain tissue by a two-step method, consisting of total EV collection first, followed by extraction of the subpopulation of microglial EVs by affinity capture with antibodies/lectins against microglial surface markers. Still, only few studies reported so far the isolation of microglia/macrophages-derived EVs from total plasma using isolectin IB4 as a myeloid marker (Scaroni et al., 2022), or from human CSF (Guo et al., 2020)/brain tissue (Cohn et al., 2021) using CD11b. Putative microglial (CD45^–^/CD11b^+^) EVs were also isolated from human tears and CSF through FACS sorting (Pieragostino et al., 2019). Other studies quantified microglial EVs in body fluids without a purification step. For example, EVs from microglia/peripheral macrophages have been measured in the CSF collected from humans or rodents by flow cytometry exploiting their positivity for IB4 (Verderio et al., 2012; Agosta et al., 2014; Joshi et al., 2014; Dalla Costa et al., 2021; Gelibter et al., 2021). Novel lab-on-chip technologies, such as ExoView (Gori et al., 2020; Skovronova et al., 2021), or Surface Plasmon Resonance (Picciolini et al., 2021), will allow further measurements and characterization of EVs from different sources and positive for myeloid (IB4, CD11b, Iba1) or specific microglial markers (TMEM119, P2Y12) once immobilized on a functionalized surface.

**FIGURE 1:**
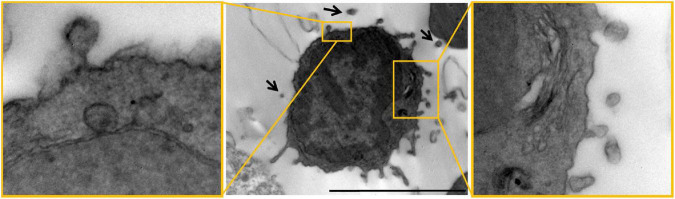
Microglial EVs imaged by electron microscopy in the mouse brain. Electron microscopy images of dark cells resembling microglia with EVs budding from their surface (“black arrows”) in the mouse corpus callosum. Scale bar 2 μm. High magnification inserts show examples of EVs budding from the surface of dark microglia. Figure modified from Lombardi et al. (2019) distributed under the terms of the Creative Commons CC BY license (Creative Commons Attribution 4.0 International License; http://creativecommons.org/licenses/by/4.0/).

Notably, EV imaging *in vivo* is still challenging (Verweij et al., 2021), especially in the brain, where the narrow conformation of the extracellular space constrains EV visualization. However, a nice example of “dark” reactive microglial cell (Bisht et al., 2016) budding EVs and surrounded by putative EVs in the pericellular space has been observed by electron microscopy (EM) at a myelin lesion site in the mouse brain (Figure 1, from Lombardi et al., 2019). Other EV-like particles, immunoreactive for the microglial receptor P2×7, have been detected by confocal analysis in mouse brain slices (Liu et al., 2017). However, no EV markers have been used to characterize these particles, and it is unclear whether the particles are detached or branch off from adjacent cells. Thus, their identification as EVs is still largely speculative. Meanwhile, the use of lipophilic dyes (e.g., PKH67, DiR/DiD, MemGlow, mCling) to label EVs brings with it a very high risk of aspecificity due to unbound dye, aggregate and EV-like micelle formation, and promiscuous labeling of non-EV particles *in vivo* as well as *in vitro* (Verweij et al., 2021). Useful strategies to image and quantify microglial EV production require the creation of transgenic mouse lines expressing fluorescent EV markers, e.g., CD9-GFP, under microglia-specific promoters or the construction of lentiviral vectors harboring the sequence of a fluorescent EV marker under the control of a microglia-specific promoter. By the latter approach, i.e., a lentivirus expressing mEmerald-CD9 fusion protein in a microglia-specific manner, in a very elegant study microglial EVs have been successfully imaged and their release quantified from single cells *in vivo* (Clayton et al., 2021). A de-targeting strategy based on miR-9, which is not expressed in microglia (Akerblom et al., 2013), was applied to avoid expression of the fluorescent EV marker in off-target cells (Clayton et al., 2021).

## Composition of microglial extracellular vesicles

Analysis of the molecular composition of microglial EVs in different conditions may help understanding their specific functions. Proteomic analysis of microglial EVs has been performed on different samples: EVs from N9 (Potolicchio et al., 2005) and BV2 (Yang et al., 2018) cell lines, primary rat microglia (Hooper et al., 2012; Drago et al., 2017; Mukherjee et al., 2020), MACS sorted rat microglia (Murgoci et al., 2020), primary leech microglia (Arab et al., 2019; Lemaire et al., 2019), microglia-like cells from human iPSCs (Mallach et al., 2021a,b; You et al., 2022), CD11b^+^ EVs from human brain tissue (Cohn et al., 2021). Their lipid (Osteikoetxea et al., 2015; Cohn et al., 2021; Kumar et al., 2021) and small non-coding RNA (miRNA) composition (Cunha et al., 2016; Prada et al., 2018; Lemaire et al., 2019; Tian et al., 2019; Cohn et al., 2021; Li et al., 2022) have also been investigated. A summary of these studies is reported in Table 2. Interestingly, only few studies performed an actual “omic” analysis of transcripts and lipids on microglial EVs (Table 2). Overall, these studies confirm the dynamic nature of microglia, revealing that EVs adapt their content to the stimuli microglia receive (Ceccarelli et al., 2021).

**TABLE 2:** Analysis of microglial extracellular vesicle composition.

Analysis	Model	References
Proteomics	Microglia-like cells from human iPSCs	Mallach et al., 2021a,b; You et al., 2022
	CD11b + EVs from human brain tissue	Cohn et al., 2021
	N9 mouse cell line	Potolicchio et al., 2005
	BV2 mouse cell line	Yang et al., 2018
	primary rat microglia	Hooper et al., 2012; Drago et al., 2017; Mukherjee et al., 2020
	CD11b/c + MACS sorted rat microglia	Arab et al., 2019; Lemaire et al., 2019; Murgoci et al., 2020
	primary leech microglia	
Transcriptomics	CD11b + EVs from human brain tissue	Cohn et al., 2021
	primary leech microglia	Lemaire et al., 2019
miRNA profiling	BV2 mouse cell line	Tian et al., 2019; Li et al., 2022
	N9 mouse cell line	Cunha et al., 2016
	primary rat microglia	Prada et al., 2018
Lipidomics	CD11b + EVs from human brain tissue	Cohn et al., 2021
Lipid analysis	BV2 mouse cell line	Osteikoetxea et al., 2015;
		Kumar et al., 2021

According to Potolicchio et al. (2005), small EVs from unstimulated N9 cells show quantitative and qualitative differences compared to other cell types, displaying a profile similar to the one of EVs from B-cells and dendritic cells. Moreover, N9-derived EVs were characterized by the presence of the aminopeptidase CD13 and the lactate transporter MCT1, which support a role for microglial EVs in neuropeptide catabolism and metabolic support to neurons respectively (Potolicchio et al., 2005).

*In vitro*, ATP stimulation of primary rat microglia induces enrichment in EVs of proteins implicated in cell adhesion/extracellular matrix reorganization, energy metabolism and autophagolysosomal pathway (Drago et al., 2017). Interestingly, the complement protein C1q, involved in synaptic pruning, is overexpressed after ATP priming too, suggesting a possible implication of microglial EVs in this process. The same study revealed that large and small EVs from primary microglia, despite displaying a set of specific proteins, share a substantial protein fraction. Primary rat microglia stimulation with Wnt3a also enriches small EVs in proteins associated with cellular metabolism, along with proteins involved in cellular architecture, protein synthesis and degradation, although no protein was detected in the constitutive EV sample (Hooper et al., 2012). In addition, BV2 cell activation with α-synuclein increases the levels of membrane-bound tumor necrosis factor (TNF) and surface MHC-II receptor in small EVs (Chang et al., 2013), while stimulation with serotonin increases their content of insulin degrading enzyme, flotillin 1 and actin (Glebov et al., 2015) and cocaine stimulation changes the expression of exosomal proteins, such as Hsps and Rab GTPases (Kumar et al., 2021).

Inflammation profoundly influences microglial EV composition (Kumar et al., 2017; Yang et al., 2018; Murgoci et al., 2020; Mallach et al., 2021a,b). In this regard LPS, a major component of the outer membrane of Gram-negative bacteria, can act in synergy with ATP, a typical danger signal released by damaged cells. In fact, only under LPS and ATP stimulation some cellular components such as GAPDH and tau are sorted in EVs by MG6 cells (Takenouchi et al., 2015) and primary microglia (Asai et al., 2015) respectively.

Under inflammation, EV release from microglia serves as unconventional secretory pathway for proinflammatory cytokines, such as IL-1β and TNF (Bianco et al., 2005, 2009; Verderio et al., 2012; Raffaele et al., 2020), and contributes to the spreading of inflammation (Verderio et al., 2012). The activation (e.g., with ATP) of so-called inflammasomes, multiprotein complexes that supervise the cytosol of immune cells and induce programmed cell-death in response to pathogens/cell damage, mediates release of EVs carrying cytokines as well as inflammasome components (Sarkar et al., 2019). EVs released upon stimulation with inflammasome activators from macrophages, the peripheral counterparts of microglia, display a specific signature (enrichment in ER- and cytoskeleton-associated genes in large and small EVs respectively) and induce opposite effects depending on the activation-state of recipient cell (Budden et al., 2021).

Differences in the molecular composition of total (i.e., large + small) EVs from differentially activated microglia, including inflammatory and pro-regenerative microglia, are clearly captured by RAMAN spectroscopy (Figure 2 from Lombardi et al., 2019), a sensitive optical technique that provides information on the chemical content of EVs (Gualerzi et al., 2021). Interestingly, RAMAN spectroscopy indicates major changes in membrane and lipid components among EV populations derived from microglia with distinct phenotypes (Lombardi et al., 2019). Lipids are structural components of EVs, but also mediate biological effects on target cells, including neurons and oligodendrocytes (Antonucci et al., 2012; Gabrielli et al., 2015; Lombardi et al., 2019). Another study confirmed changes in the lipid composition of small EVs upon microglial stimulation with cocaine (Kumar et al., 2021).

**FIGURE 2:**
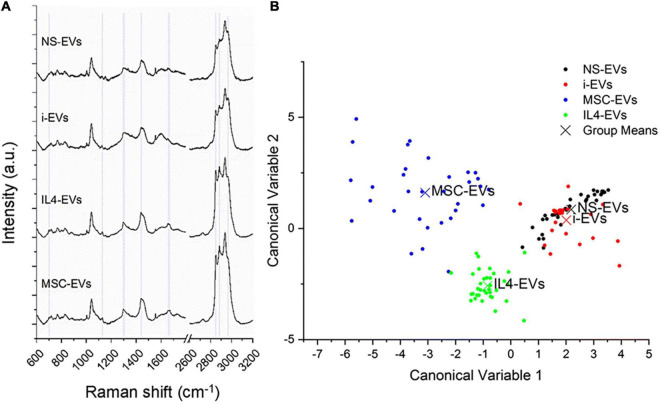
Different molecular composition of EVs from differentially activated microglia. **(A)** Mean RAMAN spectra of unstimulated (NS) or differentially polarized microglia. i-EVs are “inflammatory” EVs from microglia stimulated for 48 h with a cocktail of Th1 cytokines (20 ng/ml IL-1β, 20 ng/ml TNF-α and 25 ng/ml IFN-*γ*). IL4-EVs are from microglia stimulated with the anti-inflammatory cytokine IL-4 (20 ng/ml for 48 h). MSC-EVs are from microglia indirectly co-cultured (in transwell) with mesenchymal stem cells (MSCs) at a microglia-to-MSCs ratio of 1:1 for 48 h in the presence of Th1 cytokines. RAMAN spectroscopy is an optical technique able to provide information on the chemical content of EVs (Gualerzi et al., 2021). Spectra, obtained with a 532 nm laser line, were baseline corrected, aligned and normalized before averaging. **(B)** Multivariate statistical analysis performed on the Raman spectra (*n* ≥ 30 per sample). The scatter plot represents the values obtained for the Canonical Variable 1 and Canonical Variable 2 after LDA. In the classification model, spectra from EVs were grouped based on the cell of origin to test RS ability to discriminate the molecular composition of EVs from different microglial phenotypes. The first 10 PC scores calculated by means of PCA were used for the LDA. Each dot represents a single spectrum. Figure from Lombardi et al. (2019) distributed under the terms of the Creative Commons CC BY license (Creative Commons Attribution 4.0 International License; http://creativecommons.org/licenses/by/4.0/).

Microglia are influenced by the environment they are growing in during embryonal and early post-natal phases and maintain the differences they have acquired even after *in vitro* culturing. Murgoci et al. (2020) demonstrated that primary microglia isolated by MACS sorting from either rat cortex or spinal cord release small EVs distinct in their composition; EVs from cortical microglia are enriched in molecules related to neurite outgrowth, nerve regeneration and axogenesis pathways, while EVs from spinal microglia are enriched in molecules involved in inflammation and injury (Murgoci et al., 2020).

Small non-coding RNAs (miRNAs) are packaged into EVs, where they can travel protected from degradation, mediating a variety of EV functions (Patton et al., 2015). A few studies have analyzed the miRNA cargo of microglial EVs. Sequencing studies identified 41 miRNAs differentially expressed in small EVs isolated from IL-4 polarized BV2 cells compared to unstimulated controls, including some upregulated miRNAs (miR-23a-5p, miR-221-3p, miR-129-5p) involved in oligodendrogenesis (Li et al., 2022). Moreover, an independent study compared the miRNA profiles of small EVs from unpolarized, LPS-polarized and IL-4-polarized BV2 cells, detecting a specific enrichment of the proangiogenic miR-26a in pro-regenerative EVs with respect to the other two conditions (Tian et al., 2019). Another study showed upregulation of miR-155 and miR-146a and miR-124 downregulation in small EVs released by LPS polarized N9 microglia (Cunha et al., 2016). With respect to primary cells, Lemaire and colleagues performed RNA-seq of small EVs from resting primary leech microglia (Lemaire et al., 2019). In addition, Prada et al. profiled the miRNA composition of large and small EVs from rat primary microglia polarized toward pro-inflammatory or regenerative phenotypes or exposed to Aβ. Results from this study revealed the enrichment of 3 miRNAs (miR-146a-5p, miR-181a, and miR223) in EVs from both inflammatory and Aβ-treated cells, which silence synaptic genes and impair synaptic stability (Prada et al., 2018). To test whether the cargo of miRNAs silencing synaptic genes of EVs from DAM or inflammatory microglia may cause synaptic loss and cognitive deficits in patients with chronic inflammatory diseases, Scaroni et al. (2022) recently quantified by qPCR the expression of a set of miRNAs targeting synaptic genes in humans in IB4^+^ myeloid EVs (small EVs) isolated from the plasma of cognitively preserved and impaired patients with MS. A miRNA signature for cognitive dysfunction in MS was identified in IB4^+^ myeloid EVs, consisting of higher miR-150-5p and lower let-7b-5p (Scaroni et al., 2022).

In general, pathological conditions induce changes in the composition of microglial EVs. For example, enrichment in neurodegenerative microglial signature, consisting of increased protein expression of the DAM/MGnD markers Itgax and Apoe, has been recently detected in small EVs isolated from the brain of CAST.APP/PS1 AD mouse model (Muraoka et al., 2021). Moreover, small EVs released by microglia-like cells derived from iPSC with heterozygous R47H mutation in TREM2 (R47H*^het^* EVs), a mutation linked to late onset AD, shows alterations in cytokine/chemokine content and contain proteins linked to negative regulation of transcription and metabolic processes, in line with reported metabolic deficits of mutant microglia (Mallach et al., 2021b). A more in-depth proteomic analysis of R47H*^het^* EVs vs. EVs from subjects expressing TREM2 common variant showed that R47H*^het^* EVs reflect a different reaction of donor microglia to LPS or the TREM2 ligand phosphatidylserine (PS) (Mallach et al., 2021a). In addition, multi-omics analysis of CD11b^+^ small EVs from the parietal cortex of AD subjects vs. control and normal/low pathology cases revealed a significant reduction in the abundance of the homeostatic microglia markers P2RY12 and TMEM119, mirrored by an increase of DAM markers Ferritin Heavy Chain 1 (FTH1) and TREM2, while senescence was one of the top pathways controlled by four miRNAs found significantly upregulated in AD microglial EVs by transcriptome analysis (Cohn et al., 2021). The presence of neuron-specific, synapse-enriched and myelin-related proteins exclusively/at significantly higher levels in the proteome of AD microglial EVs reflected a hyperphagocytic microglia phenotype, in line with the increase in their lipidome of cholesterol, a major constituent of myelin (Cohn et al., 2021). Still, heterogeneity in microglial EVs emerges. In fact, not only pathology-related molecules were found in AD microglial EVs but also neuroprotective ones, indicating the presence of different EV subpopulations, either derived from the same cells or from distinct microglia, or a heterogeneous EV cargo (Cohn et al., 2021). Unexpectedly, no AD-associated changes in miRNAs involved in immune response (i.e., miR-146a-5p, miR-155-5p, and miR-124-3p; Su et al., 2016) were detected (Cohn et al., 2021), confirming the complex nature of microglial EVs.

EVs also represent a mechanism for microglia to dispose of waste products and other unwanted material, such as pathological misfolded proteins, which in fact are detected in EVs upon loading in microglia [amyloid beta (Aβ) (Joshi et al., 2014; Gouwens et al., 2018; Gabrielli et al., 2022); tau protein (Asai et al., 2015; Crotti et al., 2019); α-synuclein (Fan et al., 2019; Xia et al., 2019; Guo et al., 2020)]. This mechanism can also be exploited by pathogens (e.g., viral RNA; Luong and Olson, 2021) to move in tissues inside home-born shields.

## Insights into the biological functions of microglial extracellular vesicles

**FIGURE 3:**
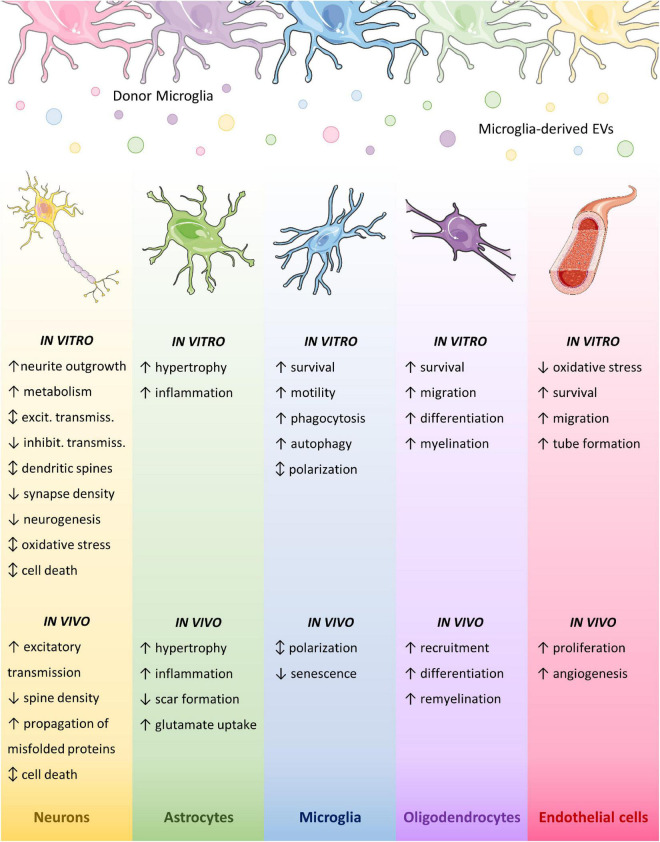
Effects of microglial EVs on brain cells. Graphic summary of microglial EV effects on brain cell types. Different colors indicate distinct activation states of donor microglia. Parts of the figures were drawn by using pictures from Servier Medical Art. Servier Medical Art by Servier is licensed under a Creative Commons Attribution 3.0 Unported License (https://creativecommons.org/licenses/by/3.0/).

Current knowledge indicates that not only microglial EVs display different functions toward other brain cells according to the activation state of donor cells, but also based on the type, or even subtype and activation phenotype, of recipient cells. In line with this, they show different interaction modalities when placed in contact with the surface of different cell types, i.e., they remain adherent to the point of contact once placed on cultured astrocytes, whilst move at the cell surface to reach the sites of internalization on microglia (Prada et al., 2016). In the following paragraphs, we describe the effects of microglial EVs on different brain cells, which are summarized in Figure 3.

### Microglial extracellular vesicle effects on neurons and synaptic function

Microglia-neuron crosstalk is essential for brain development and plasticity, while alteration in this interplay is a determinant of pathology (as reviewed by Salter and Stevens, 2017).

In physiological conditions, microglia continuously stretch and retract their fine processes toward axons and dendritic spines (Davalos et al., 2005; Nimmerjahn et al., 2005), and change their motility in response to synaptic activity and released neurotransmitters, with ATP acting as a major chemoattractant (Davalos et al., 2005; Fontainhas et al., 2011; Li et al., 2012; Abiega et al., 2016). A recent study reported that microglia respond to neuronal activation by suppressing synaptic transmission, similarly to inhibitory synapses, to protect the brain from excessive activation (Badimon et al., 2020). This is in line with the large body of evidence attesting that microglial cells work as a fundamental regulator of synaptic function (Kato et al., 2016; Acharjee et al., 2018; Basilico et al., 2022), synaptic plasticity (Schafer et al., 2013; George et al., 2016; Sipe et al., 2016; Zhou et al., 2019) and learning and memory (Rogers et al., 2011; Parkhurst et al., 2013; Cornell et al., 2022). Microglia also provide neurotrophic support to neurons and play key roles in adult neurogenesis (Sierra et al., 2010; Bachstetter et al., 2011; Gemma and Bachstetter, 2013; Marinelli et al., 2019). On the other hand, chronically activated microglia, through secretion of cytokines and other factors [glutamate, reactive oxygen species (ROS), nitric oxide (NO)], are able to induce aberrant synaptic activity and excitotoxicity (Piani and Fontana, 1994; Centonze et al., 2010; Wyss-Coray and Rogers, 2012; Vezzani and Viviani, 2015).

By eliminating supernumerary pre- and post-synaptic structures through a process called synaptic pruning, microglia are also involved in synaptic refinement and neuronal network generation during development (Tremblay et al., 2010; Paolicelli et al., 2011; Schafer et al., 2012; Weinhard et al., 2018a). Aberrant synaptic pruning during development may lead to neurodevelopmental disorders (e.g., autism, schizophrenia) (Zhan et al., 2014; Neniskyte and Gross, 2017; Salter and Stevens, 2017), while in adulthood it contributes to pathological processes in AD, Frontotemporal Dementia, MS and other neurodegenerative disorders (Hong et al., 2016; Lui et al., 2016; Werneburg et al., 2020; Lall et al., 2021). Microglia do not only eliminate synapses, but also engulf whole neurons that undergo programmed cell death (Marin-Teva et al., 2011; Brown and Neher, 2014). On the contrary, in response to specific stimuli or under chronic inflammation, microglia have also the capacity to damage and kill functional neurons (Hickman et al., 2018).

#### Microglial extracellular vesicles and neuronal development

In line with developmental microglia–neuron crosstalk, EVs from rat and leech microglia carry neurotrophic factors, such as prosaposin (Drago et al., 2017; Arab et al., 2019), and support neurite outgrowth in dorsal root ganglion (DRG) neurons (Murgoci et al., 2020), leech (Raffo-Romero et al., 2018; Arab et al., 2019), rat neurons (Lemaire et al., 2019) and PC12 cells (model for neuronal differentiation; Raffo-Romero et al., 2018). A similar effect was observed also for small EVs from iPSC-derived microglia on iPSC-derived neurons (Mallach et al., 2021a). Notably, the entity of EV action on neurites was found to be dependent on the origin of donor microglia (cortex vs. spinal cord) and the activation stimuli (e.g., LPS) (Murgoci et al., 2020).

Neurite outgrowth induced by leech microglial small EVs was associated to increased expression of proteins related to neuron development, dendrite development, axon guidance, and filopodium assembly, in recipient rat neurons (Lemaire et al., 2019). The nervous growth/differentiation factor (nGDF) of the transforming growth factor beta (TGF-β) family was implicated in this process (Raffo-Romero et al., 2018). However, it has to be pointed out that leech microglia exert specific regenerative functions, that are not shared with other species.

Intriguingly, aboundance of metabolic enzymes in EVs produced by both N9 cells (enzymes necessary for anaerobic glycolysis and lactate production; Potolicchio et al., 2005) and ATP-stimulated primary microglia (Drago et al., 2017) suggests that microglial EVs may function as conveyors of energy substrates to match the enhanced energy needs for neurite outgrowth.

Finally, consistent with the formation of filopodia/spine head filopodia at the site of contacts between microglial processes and synapses (Miyamoto et al., 2016; Weinhard et al., 2018a), placement of single microglial large EVs on dendrites of cultured neurons by optical tweezers was recently shown to induce formation of filopodia/dendritic spine protrusions starting from 2 min after EV-neuron contacts (Gabrielli et al., 2022), revealing that EVs mimic parental cell function.

#### Microglial extracellular vesicles at the synapse

Like microglia, microglial EVs influence synaptic function and their effects are complex. Once acutely administrated to cultured hippocampal neurons, large EVs released *in vitro* by ATP-stimulated microglia regulate both excitatory and inhibitory basal transmission, acting on the pre-synaptic side of the synapse (Antonucci et al., 2012; Gabrielli et al., 2015; Riganti et al., 2016). However, the effects are opposite on excitatory versus inhibitory terminals, with EVs stimulating spontaneous glutamate release (Antonucci et al., 2012; Riganti et al., 2016) while downregulating *γ*-aminobutyric acid (GABA)-ergic transmission. These effects are not consequence one of the other, but result from the activation of two independent pathways in the two neuronal subtypes (Gabrielli et al., 2015). Unexpectedly, stimulation of excitatory transmission is not mediated by the cytokine cargo of microglial EVs and is independent on the activation state of donor microglia (Antonucci et al., 2012). In fact, lipid component(s) of the EV membrane activate(s) sphingolipid metabolism in neurons stimulating sphingosine (Antonucci et al., 2012) and sphingosine-1-phosphate (Riganti et al., 2016) synthesis, which promote presynaptic release probability (Antonucci et al., 2012) and availability of synaptic vesicle for release (Riganti et al., 2016) respectively. The ability of activated microglia to stimulate excitatory transmission throughout large EVs has been confirmed in cingulate cortex slices from mouse brain (Marrone et al., 2017). In this case, EV release was stimulated by capsaicin through activation of transient receptor potential vanilloid type 1 (TRPV1), a central player in both inflammation and neuropathic pain (Marrone et al., 2017). Thus, stimulation of synaptic activity by microglial EVs may be involved in a variety of brain processes. The inhibitory action of microglial large EVs on GABAergic transmission is mediated by the bioactive endocannabinoid anandamide housed on EV membranes, which stimulates the endocannabinoid receptor type 1 (CB1) on pre-synaptic terminals to inhibit GABA release (Gabrielli et al., 2015). Notably, the opposite effects on excitatory and inhibitory transmission act in synergy to overall increase excitatory activity, as proved by *in vivo* electrophysiological recordings in the mouse visual cortex injected with microglial large EVs (Antonucci et al., 2012). Whether enhanced excitatory transmission works as a feedback mechanism to re-establish homeostasis in case of diminished neuronal activity, or drives pathological excitation-inhibition unbalance in the brain is a yet unanswered question (as reviewed in Gabrielli and Verderio, 2015).

A recent study proved that, under chronic inflammatory conditions, reactive microglia also cause post-synaptic alterations through secretion of large EVs (Prada et al., 2018). Upon prolonged neuron exposure to large EVs (72 h exposure, three EV additions, one every 24 h *in vitro*; 4 days administration in the CA1 mouse hippocampus) synapse destabilization was induced by EV-neuron transfer of a microRNA (miR-146a-5p) enriched in EVs released by inflammatory microglia (polarized with Th1 cytokine cocktail and stimulated with ATP), which silences a postsynaptic protein (neuroligin I), that is fundamental for dendritic spine formation and stability. Decreased spine and synapse density translated into impaired synapse strength, as expressed by significantly diminished miniature excitatory post-synaptic current (mEPSC) frequency and amplitude in hippocampal cultures. A similar destabilization of synapses with effects on spine remodeling is mediated by small EVs released by primary mouse microglia inflamed after saturated fatty acid palmitate exposure, a model of high-fat diet (Vinuesa et al., 2019).

Overall, the studies described above demonstrate that, at the synapse, microglial EVs can exert: (i) differential actions, mediated by distinct effector molecules (sphingolipids vs. endocannabinoids), on different neuronal subtypes (excitatory vs. inhibitory neurons), both causing, however, an excitation/inhibition unbalance; (ii) same action, mediated by different effectors (sphingosine, sphingosine-1-phosphate), on the same neuronal subtype (excitatory neurons); (iii) different actions (synaptic strength potentiation vs. decrease), mediated by different effectors (sphingosine and sphingosine-1-phosphate vs. miRNAs) on the same neuronal subtype (excitatory neurons) in different conditions, i.e., homeostatic-like vs. chronic inflammatory conditions.

#### Microglial extracellular vesicle effects on neurons in pathology

Microglial EVs have been implicated in neuronal damage in several experimental models of human pathologies (depression, glaucoma, alcoholism), or pathological risk factors (PM2.5, R47H^het^ TREM2 variant) linked to microglia inflammatory activation.

miR-146a-5p, released in EVs upon microglia inflammation, not only induces synapse destabilization (Prada et al., 2018) but also affects neurogenesis by targeting Krüppel-like factor 4 (KLF 4), as was observed in the dentate gyrus of the hippocampus of a rat model of depression (i.e., chronic unpredictable mild stress; Fan et al., 2022). Furthermore, small EVs from retinal microglia or BV2 subjected to elevated hydrostatic pressure (EHP) to mimic glaucoma (Aires et al., 2020), as well as olfactory bulb cells or BV2 exposed to particulate matter (PM2.5; Chen et al., 2020), induce oxidative stress and cell death in neurons. In the first case, the molecular mechanisms are yet to be defined (Aires et al., 2020), while in the latter the reported effects are proposed to be mediated by the presence inside EVs of the enzyme glutaminase, able to mediate glutamate production and consequent neurotoxicity (Chen et al., 2020). Ethanol activates microglia too, which subsequently release the miRNA let-7b inside large EVs together with the danger signaling molecule high mobility group box 1 (HMGB1), whose interaction is able to induce toll-like receptor (TLR) 7-mediated neurodegeneration in neurons (Coleman et al., 2017). Similarly, microglial small EVs contribute to ethanol-induced cell death of β-endorphin-producing proopiomelanocortin neurons of the hypothalamus in a rat model of fetal alcohol spectrum disorders, by spreading apoptotic factors, including reactive super-oxygen species and the complement protein C1q, part of the complement membrane attack complex (Mukherjee et al., 2020). Other studies highlight the presence in microglial EVs of the complement factor C1q (Drago et al., 2017; Lombardi et al., 2019), recently brought under the spotlights for its pivotal role in synaptic pruning, supporting a possible involvement of EVs in pathological stripping of synapses.

As mentioned in the EV composition section, small EVs released by microglia-like cells differentiated from iPSCs from patients carrying or not R47H^het^ variant of TREM2 (R47H^het^ EVs), linked to late onset AD, have been recently investigated (Mallach et al., 2021b). R47H^het^ EVs were shown to contain more inflammatory and DAM-associated proteins than common variant EVs (Cv EVs) (Mallach et al., 2021a) and to lose their protective function against oxidative stress in SH-SY5Y neurons. These findings suggest that the R47H^het^ variant may affect the ability of human microglia to protect neurons against insults in AD (Mallach et al., 2021b). Furthermore, R47H^het^ EVs were shown to be less able to promote neurite outgrowth and metabolism (reflected by increased ATP levels) in iPSC-derived neuron-like cells and SH-SY5Y (Mallach et al., 2021a) compared to Cv EVs, especially when donor microglia were treated with the TREM2 ligand phosphatidylserine (Mallach et al., 2021a). However, further analysis would be necessary to identify the molecular mechanisms responsible for impaired activity of R47H^het^ small EVs.

In neurodegenerative diseases, accumulating evidence indicates that packaging of misfolded proteins inside large and small EVs, including those of microglia origin, favors their dissemination in the brain (Joshi et al., 2014; Asai et al., 2015; Crotti et al., 2019; Guo et al., 2020; Gabrielli et al., 2022). Indeed, misfolded proteins associated to late onset neurodegenerative diseases (aβ, tau, α-syn) would not easily travel throughout the brain as insoluble aggregates, still they spread from neuron to neuron in the affected brain (Jucker and Walker, 2013; Guo and Lee, 2014). EVs prevent degradation and promote uptake/interaction of misfolded proteins with neurons (Asai et al., 2015; Gabrielli et al., 2022). In addition, EVs can enhance the pathogenic action of misfolded proteins, which become neurotoxic at a lower concentration in the lipid membrane environment of EVs (Joshi et al., 2014; Gabrielli et al., 2022).

Both Aβ and tau, the two neurotoxic proteins hallmark of AD, and α-synuclein, hallmark of PD, have been found in myeloid EVs, released from microglia/macrophages, isolated from the CSF of AD/PD patients (Saman et al., 2012; Joshi et al., 2014; Guo et al., 2020).

Aβ is taken up from the extracellular medium by microglia *in vitro* and then sorted into EVs in toxic forms, especially when intracellular degradative pathways are saturated or inhibited (Joshi et al., 2014; Gabrielli et al., 2022). Large microglial EVs carrying Aβ were originally reported by our research group to induce synaptic loss and cell death in hippocampal neurons in primary cultures (Joshi et al., 2014). More recently, it has been shown that one single Aβ-carrying large EV, gently placed in contact with the neuronal surface by optical manipulation, is able to alter dendritic spine density and maturation at the EV-neuron contact sites, while Aβ-storing large EV administration at subtoxic concentration affects synaptic plasticity (Gabrielli et al., 2022). Once injected in the mouse entorhinal cortex, a region primarily affected in AD, large EVs carrying Aβ were not only able to impair long-term potentiation (LTP), a form of synaptic plasticity that is crucial for learning and memory, *in situ*, but also to spread LTP impairment along the enthorinal-hippocampal circuit (Gabrielli et al., 2022), a key site for memory formation. Importantly, neither naked Aβ nor EVs devoid of Aβ propagated LTP impairment *in vivo* (Gabrielli et al., 2022), unveiling the essential role of large EVs carrying Aβ in the spreading of synaptic dysfunction, an early mechanism affected in AD, preceding pathological aggregates formation.

The research group coordinated by Dr. Ikezu extensively studied the role of microglial EVs in the propagation of tau and related pathology (Asai et al., 2015; Ruan et al., 2020; Clayton et al., 2021). In a seminal paper, Asai and colleagues provided evidence that microglia, through the release of EVs, are primarily involved in the propagation of tau in the mouse brain (Asai et al., 2015). Taking advantage of a novel adeno-associated virus (AAV) based model exhibiting rapid tau propagation and the P301S mouse model of tauopathy, they proved that microglial EVs propagate tau in the enthorinal-hippocampal circuit, mediating a reduction of neuron excitability and an increase in neuron apoptosis, while either microglia depletion or inhibition of EV release suppressed tau propagation (Asai et al., 2015). On this basis, they suggested that microglia may phagocyte tau-containing cytopathic neurons/synapses and secrete tau in EVs, which in turn transfer tau to other neurons. Injection of small EVs carrying tau or tau alone in wild-type mouse brain, revealed that microglial EVs transfer tau to neurons more efficiently than naked tau (Asai et al., 2015). These data were confirmed by a subsequent study showing that administration of the P2×7 ATP receptor antagonist GSK1482160, which inhibits EV secretion from microglia, blocked tau propagation in the P301S tauopathy mouse model (Ruan et al., 2020). Importantly, this treatment rescued working and contextual memory impairment in transgenic mice, indicating a suppression of disease phenotype. Finally, the same group recently described that Aβ plaques exacerbate tau propagation via microglial EVs, in an AAV-tau propagation mouse model over an AppNL-G-F genetic background (Clayton et al., 2021). In these mice, the release of tau-carrying EVs was higher from MGnD surrounding amyloid plaques that phagocyte hyper-phosphorylated plaque-associated tau as well as apoptotic neurons and synapses.

Similar to Aβ and tau protein, α-synuclein forms aggregates and is the major component of abnormal protein aggregates called Lewy bodies, which characterize PD together with loss of dopaminergic neurons in the substantia nigra. α-Synuclein is present in microglial small EVs, produced *in vitro* or isolated from the CSF of PD patients, and propagates from microglia to neurons (Guo et al., 2020). Specifically, primary microglia treated with human pre-formed α-synuclein fibrils become activated and release an increased number of small EVs enriched in α-synuclein compared to untreated cells (Guo et al., 2020). Both α-synuclein-enriched microglial EVs and CD11b-positive myeloid EVs isolated from the CSF of PD patients are taken up by neurons *in vitro*, where they induce further α-synuclein aggregation, a process enhanced by pro-inflammatory cytokines (Guo et al., 2020). The increase in small EV release and α-synuclein content is the result of α-synuclein-induced impairment of autophagy in microglia (Fan et al., 2019). In fact, α-synuclein transfer to neurons and related seeding effects can be reduced by enhancing the autophagy flux in microglia, which, in turn, inhibits EV release (Fan et al., 2019). Accordingly, a previous work showed that, upon internalization of α-synuclein storing small EVs from the plasma of PD patients, BV2 microglia exhibit an impaired autophagy flux and hypersecrete α-synuclein-carrying small EVs that, in turn, stimulate aggregation and phosphorylation of α-synuclein in SH-SY5Y neuronal cell lines (Xia et al., 2019). Once injected into the striatum of healthy mice, small EVs carrying α-synuclein cause aggregation of phosphorylated α-synuclein at the injection site and in anatomically interconnected regions, demonstrating spreading of the misfolded protein *in vivo* (Guo et al., 2020). Importantly, mice injected with α-synuclein-enriched small EVs exhibit dopaminergic neuron loss in the nigrostriatal pathway 6-months after the injection and movement disorders, pathological signs of PD not found in the sham group (Guo et al., 2020). In line with this study, small EVs from BV2 microglia and carrying α-synuclein were previously shown to induce cytotoxicity and apoptosis in neurons *in vitro* (Chang et al., 2013).

Interestingly, microglial EVs may also exert beneficial effects in pathology, frequently mediated by their miRNA cargo. For example, vesicular miR-124 has been shown to be beneficial in different pathological contexts. Relatively to ischemic stroke, pro-regenerative BV2 microglia (exposed to IL-4 for 48 h) release small EVs containing miR-124, which protects neurons from apoptosis after oxygen-glucose deprivation, and *in vivo* reduces infarct volume and ameliorates behavioral deficits after transient middle cerebral artery occlusion (MCAO), having ubiquitin-specific protease 14 as target (Song et al., 2019). In traumatic brain injury (TBI), a condition that drastically increases the risk to develop neurodegenerative diseases included AD, miR-124 is overexpressed in microglia, promoting their polarization toward a pro-regenerative phenotype, and is released inside EVs (Huang S. et al., 2018; Ge et al., 2020). miR-124 in microglial small EVs, through the suppression of PDE4B targeting mTOR signaling, protects against neurodegeneration, inhibiting neuronal inflammation and expression of RhoA and neurodegenerative proteins (Aβ and phospho-tau) and mediating neurite outgrowth, 72 h after scratch injury (Huang S. et al., 2018). Accordingly, intravenous injection of small EVs, from miR-124-3p-overexpressing BV2 cells, improves mice cognitive outcome in a TBI mouse model by targeting Aβ production through the Rela/ApoE signaling pathway (Ge et al., 2020). Vesicular miR-711 has also been found to be effective in TBI. In fact, small EVs released by BV2 cells, skewed toward a pro-regenerative phenotype by miR-711 overexpression through 1,4,5-trisphosphate 3-kinase B (Itpkb) silencing, reduce neurological deficits and improve cognitive function upon injection into mice subjected to TBI (Zhang et al., 2020). Finally, miR-135a-5p is another protective miRNA of small EVs derived from pro-regenerative BV2 cells. It was reported to reduce neuronal autophagy and ischemic brain injury in a mouse model of transient MCAO by inhibiting nod-like receptor protein 3 (NLRP3) inflammasome through thioredoxin-interacting protein (TXNIP) (Liu et al., 2021).

#### Microglial extracellular vesicle-neuron interaction

How microglial EVs interact with neurons to deliver and/or propagate pathological signals in the brain is currently under intense investigation. It has been observed through co-culture and microfluidic approaches that small EVs from mixed brain cells or neurons can be internalized by neurons and move inside axons to trans-synaptically transfer their cargo (Wang et al., 2017; Polanco et al., 2018; Sardar Sinha et al., 2018). This is likely to happen for microglial small EVs too, although only data supporting internalization into neurons (Song et al., 2019; Guo et al., 2020; Mallach et al., 2021b) but not intracellular transport along axons, have been reported so far for this subpopulation. Intriguingly, a new study reported that large microglial EVs, gently placed on the neuronal membrane of cultured neurons by optical manipulation, can efficiently move at the surface of neurons and, in particular, along axonal projections, without being internalized (Gabrielli et al., 2022), as already demonstrated for large EVs derived from astrocytes (D’Arrigo et al., 2021). Importantly, large EVs carrying Aβ are more prone to motility along axons, move faster and in a prevalent anterograde direction compared to EVs devoid of Aβ (Gabrielli et al., 2022). This suggests that microglia priming with Aβ drives the sorting into large EVs of molecules stimulating extracellular EV motion at the neuron surface. During their path along axonal projections, large EVs may undergo a transient fusion with the neuron plasma membrane (Prada et al., 2018), allowing direct transfer of genetic material to the neuron cytoplasm. Large EVs moving along neurites intensively scan the neuronal surface and often stop at preferential sites, especially on mature dendrites. Further analysis will be necessary to explore the fascinating hypothesis that these preferential sites may be in fact synaptic boutons.

**FIGURE 4:**
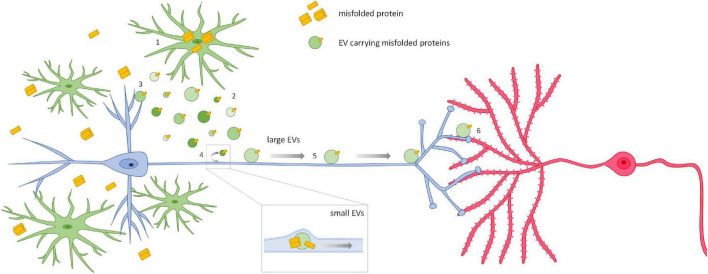
Spreading of neurodegenerative signals via microglial EVs. The current opinion is that, when misfolded proteins accumulate in the affected brain, microglia internalize them from the extracellular space (1), or by phagocyting dying cells or cell debris, and then release the proteins in association with EVs (2). This process is amplified when intracellular degradative pathways are saturated or compromised. EVs carrying neurotoxic proteins affect neurons and synaptic transmission at the site of release (3). However, EVs are also able to propagate their pathogenic signals among neurons. Two mechanisms for EV-mediated trans-synaptic propagation of misfolded proteins have been described so far. Small EVs isolated from human and murine brains or released by primary neurons can be internalized by neurons and travel inside axons to trans-synaptically transfer their misfolded cargo (4) (Wang et al., 2017; Polanco et al., 2018; Sardar Sinha et al., 2018). This may well happen for small EVs released by microglia. On the other hand, microglial large EVs, too big to be transported intracellularly, move at the axonal surface using neuronal processes as highways (5) to deliver signals to connected cells (6) (Gabrielli et al., 2022). These mechanisms may be involved in the propagation of other pathological mediators in neurodegenerative diseases characterized by *trans*-neuronal propagation.

The molecular mechanisms underlying microglial EV-neuron interaction and the dynamics of both intracellular and extracellular motion along neuronal processes (Figure 4) are still largely obscure. Clarifying these mechanisms will be fundamental in order to find new targets for the development of novel therapeutic strategies to hamper the progression of neurodegenerative diseases spreading along neuronal connections.

### Microglial extracellular vesicle effects on astrocytes

The interaction between microglia and astrocytes significantly influences neuroinflammation (Liu et al., 2020). In particular, during CNS insult or injury, microglia-derived signals are key determinants for the phenotype and function of astrocytes (Liddelow et al., 2017; Hasel et al., 2021). Emerging evidence indicates a crucial involvement of EVs in microglia-astrocyte interaction. Large EVs derived from inflammatory microglia, activated *in vitro* with LPS and/or inflammatory cytokines, were originally reported to propagate an inflammatory response to cultured astrocytes, which became hypertrophic and upregulated inflammatory markers (Verderio et al., 2012).

In a subsequent study, total (large + small) EVs released by inflammatory primary rat microglia were shown to carry the three mediators that are necessary and sufficient to transform astrocytes toward A1 harmful cells, i.e., IL-1a, C1q, and TNF (Liddelow et al., 2017), and to induce A1 astrocyte conversion both *in vitro* and at focal myelin lesions (Lombardi et al., 2019). Harmful astrocyte transformation in response to microglial EVs was indicated by analysis of the expression and immunoreactivity of a few markers of protective (PTX3, CD14, and Tm4sf1) and detrimental (serping-1, amigo-2, C3) astrocytes. The study also provided first insights on the molecular components of microglial EVs involved in detrimental (and neuroprotective) astrocyte transition. Indeed, inactivation of vesicular TNF cargo by the TNF inhibitor Etanercept partially counteracted A1 astrocyte activation *in vivo* while shifting cultured astrocytes toward A2 protective phenotype. While confirming the involvement of TNF in A1 harmful astrocyte transformation, these data also highlighted the presence of protective molecules in EVs released by inflammatory microglia. Interestingly, in EV fractionation experiments, protective molecules were recovered in the lipid extracts of microglial EVs, pointing to the relevance of lipids released by microglia via EVs in pro-regenerative research. Overall, this study indicates that microglial EVs contain well-known proteins mediators involved in detrimental astrocyte transition but also bioactive lipids promoting protective astrocyte transition as well as oligodendrocyte precursor cell differentiation (see section “Microglial EV effects on oligodendrocytes and remyelination”).

Consistent with a dominant inflammatory activity of EV protein cargo, another study showed that neither lipids extracted from microglial large EVs nor broken large EVs, depleted by their luminal cargo, increase pro-inflammatory transcripts in recipient astrocytes (Drago et al., 2017). This work also showed that contact between astrocytes and EVs is necessary for astrocyte activation.

In stroke, the interaction between microglia and astrocytes is pivotal for the regulation of glial scar formation, a major obstacle for axonal regeneration and functional recovery (Grimpe and Silver, 2004; Silver and Miller, 2004). The group of Drs. Zhang and Yang found that small EVs from IL-4 polarized BV2 cells are able to attenuate glial scar formation after transient MCAO by transferring to astrocytes miR-124, a miRNA targeting the astrocyte activating molecule STAT3. miR-124 transfer via EVs seems also to induce a transition of reactive astrocyte to neuronal progenitors and to reduce mouse brain atrophy volume, improving neurobehavioral outcomes after ischemia (Li et al., 2021).

Interestingly, small EVs from BV2 cells have also been reported to reduce glioma growth and toxicity in a dose-dependent way, regardless of donor cell activation state (Serpe et al., 2021). The EVs act on cell metabolism on both glioma cells and surrounding astrocytes, which are enhancers of tumor colonization (Fitzgerald et al., 2008; Rath et al., 2013). They reduce astrogliosis (GFAP staining) in the peritumoral region and increase the expression of the glutamate transporters Glt-1 and Glast on astrocytes to enhance glutamate uptake, thus reducing excitotoxicity. Again, the beneficial effects of EVs are mediated by their cargo of miR-124, which acts as a tumor suppressor (Huang J. et al., 2018).

### Microglial extracellular vesicle effects on microglia functional phenotype

Microglia are extremely plastic cells, able to react to CNS insults by acquiring multiple functional states to favor tissue remodeling or degeneration (Stratoulias et al., 2019). The activation of microglia is a transitory process, which progressively declines to restore tissue homeostasis if not sustained by continuous stimuli coming from the environment. However, microglia have also the capacity to self-sustain their activation in a positive feedback loop, by releasing cytokines and growth factors acting in an autocrine fashion (Kuno et al., 2005; Zhang et al., 2014) or to self-limit their response by producing anti-inflammatory cytokines, like IL-19 (Horiuchi et al., 2015).

Recent evidence shows that microglia can capture and internalize EVs produced by other microglial cells, suggesting the possibility that different microglial phenotypes may influence each other through EV release (Casella et al., 2018; Van den Broek et al., 2020; Lecuyer et al., 2021). Importantly, activated microglia isolated from ischemic brain regions showed a greater capacity of EV uptake than resting cells of the contralateral side, indicating that the functional state of recipient cells influences their capacity of interacting with EVs (Lecuyer et al., 2021). Following internalization, small EVs derived from BV2 cells can play a beneficial role on microglia under inflammatory stress, by modulating protective activities such as cellular movement, cell death and survival, as well as cellular growth and proliferation (Van den Broek et al., 2020). Results from a recent study also suggest that small EVs released by microglia may play a fundamental role in regulating the phagocytic capacity of microglia (Huang et al., 2022). Specifically, the phagocytosis receptor TREM2 has been detected on the surface of small EVs from BV2 cells, where it mediates the binding of EVs to Aβ fragments. Such EVs-Aβ interaction was found to alter the inflammatory microenvironment around protein aggregates, increasing the local concentration of chemokines which in turn facilitate the recognition and engulfment of Aβ fragments by microglia (Huang et al., 2022). Regarding the intracellular responses evoked by microglial small EVs, recent data show that internalization in cultured unstimulated and TNF-activated microglia is followed by a significant upregulation of the autophagy marker LC3B-II and a consequent increase of the autophagic flux (Van den Broek et al., 2020). Moreover, transcriptomics experiments demonstrated that microglial small EVs induce a significant downregulation of key apoptotic and inflammatory genes in unstimulated or TNF-activated recipient microglia (Van den Broek et al., 2020). Therefore, microglial EVs, by an autocrine feedback, may play an important role in the early response of microglia to tissue damage and subsequent cellular stress, inhibiting cell death pathways in favor of survival signaling and autophagy to preserve cell homeostasis and functionality (Van den Broek et al., 2020).

The immunomodulatory role of microglial EVs appears to be also strictly dependent on the phenotype of donor cells. When administered *in vivo*, total (large + small) EVs derived from pro-inflammatory microglia significantly enhanced the expression of inflammatory markers in microglial cells populating the boundaries of ischemic brain lesions (Raffaele et al., 2021). Moreover, pharmacological blockade of endogenous microglial small EV release, using a neutral sphingomyelinase inhibitor, significantly counteracted microglia pro-inflammatory activation around the ischemic lesion and increased the expression of pro-regenerative markers (Gao et al., 2020). In another report, pro-inflammatory large EVs were found to promote a phenotypic switch of glioma-associated microglia toward an inflammatory profile, significantly reducing tumor growth in mice (Grimaldi et al., 2019). Small EVs released by microglia exposed to elevated hydrostatic pressure (EHP), mimicking elevated intraocular pressure in glaucoma, favored detrimental functions of retinal microglia both *in vitro* and upon intravitreal injection *in vivo*. This harmful phenotype induced by EVs was characterized by increased production of pro-inflammatory cytokines, motility, phagocytic efficiency, and proliferation (Aires et al., 2020). Furthermore, divalent manganese (Mn^2+^), a transition metal whose occupational exposure is associated with increased risk of neurodegenerative diseases in miners and welders, increased the activation of NLRP3 inflammasome and induced cell-to-cell transfer of the inflammasome adaptor protein ASC via small EVs (Sarkar et al., 2019). In response to stimulation with Mn^2+^-induced small EVs, primary microglia increased IL-1β production, suggesting a role for microglial EVs in expanding inflammasome cascades following exposure to toxic environmental stimuli (Sarkar et al., 2019). These data are in line with previous studies proposing a role for microglial EVs in spreading inflammatory signals within the injured CNS, amplifying detrimental responses originated at the site of lesion (Verderio et al., 2012).

Conversely, pro-regenerative microglia-derived EVs were shown to significantly foster the resolving properties of recipient microglial cells. In a murine model of brain ischemia, total EVs obtained from primary microglia stimulated with the anti-inflammatory IL-4 promoted a shift toward a pro-regenerative phenotype of recipient pro-inflammatory and dystrophic microglial cells at the boundary of the ischemic lesion. In detail, recipient microglia exhibited an increased expression of the protective marker Ym1 and morphological rearrangements, indicative of a recovery from a senescent-like phenotype to a functional one (Raffaele et al., 2021). Another independent study showed that treatment of ischemic mice with EVs isolated from hypoxia-preconditioned microglia, an experimental condition characterized by pro-regenerative traits, significantly elevated the number of microglial cells expressing the regenerative marker CD206 via delivery of TGF-β1 (Zhang et al., 2021). Since aberrant microglia activation represent a common pathogenetic mechanism to different neurological disorders, the identification of the molecular components responsible for the beneficial effects of pro-regenerative EVs on microglial phenotype might provide novel candidate targets to sustain the regenerative functions of microglia during CNS repair.

Finally, engineered microglial EVs may also represent a biological drug delivery platform to shape the activation state of resident microglial cells at lesion sites. In this respect, pro-regenerative total EVs isolated from an engineered microglial cell line, overexpressing the endogenous “eat me” signal Lactadherin (Mfg-e8) on the surface to target phagocytes and enriched in IL-4, were specifically uptaken by microglial cells in the EAE model of MS (Casella et al., 2018). Once internalized, IL-4 loaded EVs favored the upregulation of anti-inflammatory markers Ym1 and Arg1 in recipient cells, significantly reducing tissue damage and clinical symptoms (Casella et al., 2018). Similarly, microglial small EVs enriched in anti-inflammatory miR-711 were shown to reduce inflammation and cognitive dysfunction in a TBI model by promoting a protective phenotype of resident immune cells (Zhang et al., 2020).

### Microglial extracellular vesicle effects on oligodendrocytes and remyelination

An increasing body of evidence indicates the importance of the functional interaction of microglial cells with oligodendrocytes, the myelin-forming glial cells of the CNS, for myelin development, maintenance, and regeneration after damage. During development, microglia orchestrate oligodendrogenesis and myelination, by inducing oligodendrocytes precursor cell (OPC) differentiation and axon ensheathing (Hagemeyer et al., 2017) and by removing excessive and ectopic myelinated tracts, with a mechanism resembling synaptic pruning (Hughes and Appel, 2020). These immune cells also tightly regulate the number of quiescent OPCs in the adult white matter (Hagemeyer et al., 2017).

Previous studies have shown that the proper interplay between microglia and oligodendrocytes is also essential for efficient remyelination. In fact, in different experimental models of CNS degeneration, myelin regeneration impairment occurs when microglia are removed or their action blocked (Li et al., 2005; Miron et al., 2013; Tanaka et al., 2013; Pavic et al., 2021; Raffaele et al., 2021). This also happens during aging, when defective myelin turnover has been correlated with a reduction in the recruitment of microglia, in their activation toward a pro-regenerative phenotype, and in their phagocytic potential (Rawji et al., 2016).

The regenerative functions of activated microglia in response to a demyelinating insult have been ascribed to their capability to phagocytose myelin debris, secrete protective factors, and modulate the extracellular matrix composition, thus creating an environment that supports the recruitment of OPCs and their subsequent differentiation into myelinating oligodendrocytes (Lloyd and Miron, 2019). Importantly, at late injury phase, the pro-remyelinating features of microglia are lost and replaced by a dystrophic senescent-like phenotype, characterized by deficits in phagocytic function and the production of pro-inflammatory molecules hindering efficient myelin repair (Rawji et al., 2020; Raffaele et al., 2021). Elucidating the mechanisms involved in microglia-OPC crosstalk may reveal novel strategies to limit myelin damage and enhance remyelination in the CNS.

Noteworthy, microglial cells infiltrating demyelinated lesions were found to secrete large amounts of EVs, suggesting that EVs may represent fundamental players in the communication between microglia and oligodendrocyte lineage cells (Lombardi et al., 2019). Recently, the effects of EVs produced *in vitro* by either pro-inflammatory or pro-regenerative microglia on OPCs at demyelinated and ischemic lesions have been described (Lombardi et al., 2019; Raffaele et al., 2021). Infusion of pro-regenerative EVs (large and small EVs released by microglia polarized with IL-4 *in vitro* or cocultured with immunomodulatory mesenchymal stem cells) at lysolecithin-induced focal myelin lesion significantly promoted OPC migration and differentiation, resulting in improved remyelination. Conversely, in this experimental setting, EVs shed by pro-inflammatory cells (stimulated with Th1 cytokines) inhibited remyelination (Lombardi et al., 2019). By using a model of experimental stroke induced by MCAO, we also analyzed the effects induced by microglial EVs on GPR17-expressing OPCs, a cluster of oligodendrocytes particularly reactive to brain damage (Lecca et al., 2020). Infusion of pro-regenerative microglial EVs in the ipsilateral corpus callosum of ischemic mice starting from day 14 post-MCAO, corresponding to the late stage after stroke, specifically increased the number of GPR17-expressing OPCs at lesion boundaries and enhanced their maturation (Raffaele et al., 2021). Accordingly, EVs obtained from pro-regenerative microglia significantly improved myelin integrity in the ipsilateral corpus callosum, suggesting that efficient remyelination occurred. On the other end, in this model, the infusion of pro-inflammatory EVs did not induce significant changes in the number of GPR17-expressing OPCs recruited at lesion borders nor in their degree of maturation (Raffaele et al., 2021). These results have been recently confirmed by another independent study, showing that intravenous delivery of pro-regenerative small EVs isolated from IL-4 polarized BV2 cells significantly promoted white matter structural remodeling and remyelination after transient MCAO (Li et al., 2022).

Hence, these *in vivo* studies strongly indicate that pro-regenerative microglial EVs always exert beneficial effects on OPC response to myelin damage, while the action of pro-inflammatory EVs on oligodendrocytes may be highly dependent on the activation state of astrocytes. In this respect, further analyses carried out in the lysolecithin model showed that the block of remyelination caused by pro-inflammatory EVs mostly depends on the astrocyte transformation into harmful cells, rather than a direct effect on oligodendrocytes (Lombardi et al., 2019). Astroglial functions are known to depend on the nature of the damaging stimulus (Liddelow et al., 2017; Hasel et al., 2021). While, in LPS-induced neuroinflammation, astrocytes assume a detrimental phenotype, they maintain a beneficial and protective action following experimental stroke (Zamanian et al., 2012). The heterogeneity of astrocyte response could therefore explain the detrimental versus neutral action of pro-inflammatory EVs observed in our experiments (see also section “Microglial EV effects on astrocytes”).

The identification of the molecular components responsible for the beneficial effects produced by pro-regenerative microglial EVs could reveal new pharmacological targets to be exploited for remyelinating therapies. On this basis, multiple *in vitro* systems have been employed to dissect the mechanisms underlying the harmful and beneficial actions of microglial EVs on OPCs (Lombardi et al., 2019; Raffaele et al., 2021; Li et al., 2022).

While *in vitro* assays based on EdU incorporation showed very mild effects on OPC proliferation, results unveiled a pronounced impact of microglial EVs on OPC migration, in agreement with *in vivo* data. Indeed, all tested types of microglial EVs significantly enhanced the migration of OPCs in the Boyden chamber chemotaxis systems, regardless of the activation state of donor cells (Lombardi et al., 2019). Interestingly, the chemoattractant effect of microglial EVs on OPCs was abolished by simultaneous treatment with sphingosine-1-phosphate (S1P) receptor antagonists, indicating that vesicular S1P may be involved in recruiting OPCs toward myelin lesions to initiate repair (Lombardi et al., 2019). Moreover, pro-regenerative small EVs from BV2 cells were found to significantly increase OPC survival assessed by the CCK-8 assay both in normal conditions and upon oxygen-glucose deprivation (Li et al., 2022).

*In vitro* experiments also confirmed the capacity of microglial EVs, including those released by pro-inflammatory cells, to directly enhance OPC differentiation into MBP-expressing cells and myelination of DRG neurons in co-culture. However, oligodendrocyte maturation was impaired when OPCs were exposed to pro-inflammatory EVs in the presence of astrocytes. This result suggests that astrocytes mediate the detrimental action of inflammatory microglia-derived EVs on OPC maturation, similarly to what observed *in vivo* (Lombardi et al., 2019). *In vitro* results also showed that EVs from pro-regenerative microglia specifically enhanced the maturation of the GPR17-expressing subset of OPCs, representing the pool of cells mostly committed to myelination (Lecca et al., 2020), while they have no impact on the GPR17-negative fraction of progenitors (Raffaele et al., 2021).

Some experiments have been done to investigate whether the effects of microglial EVs on OPCs are mediated by surface or cytosolic components. By exposing vesicles to osmotic shock conditions, the effects of broken EVs, depleted of their content, were compared to intact EVs. Of note, broken EVs retained the capability to promote OPC maturation, suggesting the main involvement of surface molecules in this process (Lombardi et al., 2019). In this respect, vesicular transmembrane TNF, already known to be present in myeloid vesicles (Yang et al., 2018; Raffaele et al., 2020) and to drive OPC differentiation upon activation of oligodendroglial TNFR2 (Madsen et al., 2016), has been shown to exert a role in the pro-differentiating effects of microglial EVs (Raffaele et al., 2021). Furthermore, treatment with the purified lipid fraction of microglial EVs enhanced OPC maturation even more efficiently than intact EVs, suggesting that lipid components crucially contribute to fostering OPC differentiation (Lombardi et al., 2019; Gualerzi et al., 2021). While our results rule out the involvement of S1P in the pro-differentiating effects of microglial EVs (Lombardi et al., 2019), other lipid components enriched in vesicular membranes, including endocannabinoids (Gabrielli et al., 2015) and sterols (Pfrieger and Vitale, 2018), have been already linked to OPC maturation (Ilyasov et al., 2018; Berghoff et al., 2021) and may therefore be responsible for these effects. However, the vesicular lipids regulating the pro-myelinating action of microglial EVs remain to be determined (Gualerzi et al., 2021). The pro-differentiating effects of microglial EVs may be also partly mediated by their miRNA cargo, as miRNAs have been largely implicated in regulating OPC maturation (Marangon et al., 2019). In this respect, several miRNAs enriched in pro-regenerative small EVs from BV2 cells significantly improved OPC viability and differentiation *in vitro*. Among those, knockdown of miRNA-23a-5p in donor BV2 cells was found to abolish the pro-myelinating properties of regenerative small EVs, demonstrating an essential role of this specific miRNA in the effects observed (Li et al., 2022).

Based on these data, it is reasonable to speculate that a combination of different factors with diverse biochemical nature, rather than a single molecule, should be responsible for EV-mediated beneficial effects on OPCs and remyelination. This makes exciting, albeit extremely challenging, to identify candidate compounds enriched in pro-regenerative EVs to be exploited for therapeutic purposes. Nevertheless, a different strategy could be employed to translate the beneficial effects exerted by microglial EVs into pharmacological treatments. For example, multi-omics approaches on OPCs exposed to microglial EVs may unveil relevant cellular processes to be targeted for novel remyelinating therapies.

### Microglial extracellular vesicle effects on endothelial cells and angiogenesis

By regulating cerebrovascular plasticity and blood-brain barrier (BBB) permeability, microglia also control the exchange of nutrients and immune cells between the brain and the circulation (Dudvarski Stankovic et al., 2016). During embryonic development, microglia orchestrate CNS vascularization by guiding the sprouting of growing capillaries via release of vascular endothelial growth factor (VEGF) and other proangiogenic factors (Arnold and Betsholtz, 2013). In the adult CNS, about 30% of microglial cells are associated with capillaries and constantly interact with endothelial cells and pericytes to regulate vascular diameter and cerebral blood flow (Bisht et al., 2021). Adult angiogenesis is crucial for CNS repair mechanisms following traumatic, ischemic, or degenerative insults, because revascularization ensures the trophic support necessary for tissue remodeling and maintenance (Ma et al., 2021). Moreover, the generation of new blood vessels is a key determinant of brain tumor growth and invasiveness (Dudvarski Stankovic et al., 2016). Depending on the specific pathological condition, the interaction of activated microglia with blood vessels is crucial to accelerate disease progression or to retard degeneration and start reparative mechanisms. Due to their role in cell-to-cell communication, microglia-derived EVs may represent attractive tools to manipulate angiogenesis by directly targeting endothelial cells. In this respect, a recent report showed that primary microglia-derived small EVs are efficiently internalized by endothelial cells, inducing a consequent reduction of intracellular oxidative stress levels mediated by activation of the keap1/Nrf2/HO-1 pathway (Peng et al., 2021). By this mechanism, microglial EVs improved endothelial cell function under oxidative challenge *in vitro*, increasing survival and fostering cell migration and tube formation capacity (a readout of vessel formation during angiogenesis) (Peng et al., 2021). Moreover, administration of microglial EVs significantly enhanced the proliferation and regeneration of CD31-positive vessels after contusive spinal cord injury (SCI) *in vivo*, contributing to reduced lesion volume and improved functional recovery (Peng et al., 2021). In a different work, pro-regenerative EVs obtained from hypoxia-preconditioned primary microglia significantly increased the viability, migration, and tube formation capacity of endothelial cells exposed to oxygen-glucose deprivation, an *in vitro* model of hypoxic/ischemic injury (Zhang et al., 2021). This effect was found to be mediated by activation of the TGF-β1/Smad2/3 pathway, as both combined treatment with TGF-β inhibitor and TGF-β silencing in donor microglial cells prior to EV isolation abolished the pro-angiogenic properties observed (Zhang et al., 2021). Finally, administration of hypoxia-preconditioned microglia-derived EVs *in vivo* increased the proliferation of CD31-positive endothelial cells after experimental stroke, contributing to reduced cell death in the peri-infarct region (Zhang et al., 2021). As shown for other types of acceptor cells, the effects induced by microglial EVs on endothelial cells may be dependent on the phenotype of the donor. Indeed, small EVs collected from IL-4 stimulated BV2 cells displayed pro-angiogenic properties, increasing tube formation by endothelial cells *in vitro*, while EVs obtained from LPS-stimulated cells had no effects as compared to unstimulated controls (Tian et al., 2019). In this case, the proangiogenic effect of microglial EVs was not ascribed to growth factors, whose levels were found to be comparable in all types of EVs examined, but rather to the transfer of miR-26a, that was specifically enriched in IL-4 induced EVs compared to the other experimental conditions (Tian et al., 2019).

Finally, microglial EVs can also participate to HIV-1 associated neurocognitive disorders (HAND) linked to BBB dysfunction and persistent inflammation by incorporating viral proteins (Mahajan et al., 2021). In fact, microglia transfected with Nef (a viral protein involved in HIV replication and a main determinant of viral pathogenesis) release Nef-carrying EVs that are able to disrupt the integrity of the BBB (Raymond et al., 2016). Notably, this effect was also associated with an increase in IL-12, IL-8, IL-6, RANTES, and IL-17A secretion from microglia.

## Sexual dimorphism in microglial extracellular vesicles

It is now recognized that brain development differs between male and female (McCarthy, 2016). Whether this is to be accounted to sex chromosomes and/or sex hormones is still a matter of a debate. However, sex differences in microglia function seem to play a key role not only in development, but also in pathology, including autism spectrum disorders, AD, TBI, ischemia, and stress (Schwarz et al., 2012; Arevalo et al., 2013; Acaz-Fonseca et al., 2015; Bollinger et al., 2016; McCullough et al., 2016; Hanamsagar et al., 2017). Ergo, sex can be a strong contributor to both homeostatic and pathological functions of microglia in mammals, which has to be taken into account in a precision medicine perspective. Although the exact molecular mechanisms underlying these differences are still largely unknown, recent findings suggest that microglia activation and specification into phenotypes are influenced by sex, leading to discrepancies in the inflammatory/pro-regenerative responses upon CNS insult (Acaz-Fonseca et al., 2015; Kerr et al., 2019).

Sexual dimorphism in EV composition and functions has already been reported for EVs secreted in the female reproductive organs during menopause reproductive senescence. These EVs, likely in response to an innate immune inflammatory response in the reproductive organs, shuttle inflammasome proteins to the brain, making females more susceptible to ischemic damage (Raval et al., 2019). Furthermore, a very recent study examined the effects of physical exercise, coupled to sleep deprivation and caloric restriction on blood EV size, concentration, and surface proteins in men and women, identifying sexually dimorphic EV profiles (Conkright et al., 2022), in line with previous studies reporting the influence of sex or menstrual cycle in vesicle biogenesis under specific circumstances (Toth et al., 2007; Durrer et al., 2015; Gustafson et al., 2015; Lansford et al., 2016; Rigamonti et al., 2020). EV differences between sexes have been found in invertebrates too (Harischandra et al., 2018; Goodwin and Hobert, 2021).

To the best of our knowledge, sex differences related to microglial EVs have only emerged in one study so far. Crotti et al. (2019) observed that Bridging INtegrator 1 (BIN1), an adaptor protein that regulates lipid membrane dynamics, whose gene mutation are highly associated to Late Onset AD, favors the spreading of AD pathological protein tau via EVs. However, targeted deletion of BIN1 from microglia decreased tau spreading *in vivo* only in male and not female PS19 mice. In line with these results, BIN1 deletion induced a significant downregulation of the expression of several heat-shock proteins involved in tau proteostasis in microglial cells from male but not female mice. These data indicate a sex-specific role for BIN1 in tau vesicular secretion from microglia, as well as in tau proteostasis. Further experiments are needed to address the enticing possibility that microglial EV functions may be influenced by individual’s sex.

## Discussion

Ten years ago, we already knew that EVs released by rodent microglia contain various inflammatory molecules, including IL-1β and MHC-II, and propagate an inflammatory signal to neighboring cells (microglia/astrocytes), playing a possible pathogenic role in neuroinflammatory diseases, such as MS (Verderio et al., 2012). Few important pieces of information were available on the molecular mechanisms of EV production from microglia in rodents. We knew the stimulus (ATP), the receptor (P2×7) and the key enzyme (acid sphingomyelinase) involved in large EV release from microglia (Turola et al., 2012). However, the molecular composition of microglial EVs and the bioactive agents responsible for EV effects on target cells (microglia/astrocytes/neurons) were largely unknown. Little information was also available on the modalities of EV interaction with target cells and about possible changes of EV cargo in relation to the activation state of donor microglia. Whether microglial EVs contribute to neurodegenerative diseases, where microglia involvement is proven by genome-wide association studies, was completely unexplored.

In the last decade our knowledge on microglia and the EVs they release has increased exponentially. The studies described in this review clearly indicate that the transcriptional profiles and the regional origin of microglia influence both composition and action of EVs on target cells, which now include oligodendrocytes and endothelial cells in addition to microglia, astrocytes and neurons. Furthermore, evidence is emerging that the action of microglial EVs also depends on the activation state of recipient cells.

Multiple stimuli have been shown to activate EV production from microglia (cytokines, LPS, capsaicin, serotonin, amyloid plaque and others) besides ATP, and it is now clear that microglia dynamically modify EV composition in response to environmental stimuli, thus generating a heterogenous EV population. Omics analysis of EVs have greatly helped deciphering the message stored inside EVs and sent to the surrounding cells by either wild type and mutant microglia (e.g., microglia with R47H^het^ mutation, linked to late onset AD). Multiple bioactive components have been identified among protein, lipid, and miRNA cargoes of EVs, and proved to control neurotransmission and synapse stability (endocannabinoids, miR-146) as well as oligodendrocyte migration (S1-P) and maturation (tmTNF, miR-23a-5p) in multiple ways, including cargo transfer and activation of signaling events at the cell surface. It is worth to note that, while we have collected several proteomic and miRNA data on microglial EVs in the last 10 years, only very limited knowledge is available on the lipid and mRNA cargo of microglial EVs. Further lipidomic and transcriptomic data in the years to come will increase our understanding of microglial EVs signaling to the microenvironment and might help the design of engineered vesicles for targeted delivery of biomolecules across the CNS barriers (Banks et al., 2020), accelerating the use of EVs as therapeutic tools in neurological diseases (Mentkowski et al., 2018). However, the most important challenge we face for the next years is to develop new techniques for analysis of EV cargo at single EV resolution. This will be fundamental to explore possible heterogeneity in chemical content of EVs produced by single microglial cells.

Extensive literature has revealed that under neurodegenerative conditions microglial EVs become vehicle of misfolded proteins, including Aβ and tau, and propagate synaptic dysfunction among connected neurons both *in vitro* and *in vivo*. Intriguingly, analysis of EV-neuron interaction revealed that small and large EVs exploit different mechanisms to spread their pathogenic signals among neurons: small EVs enter the neuron cytoplasm to be transported inside axons up to the nerve ending, while large EVs move at the neurons surface and use axons as a route to reach synaptically connected neurons *in vitro*. Despite these findings suggest that also *in vivo* microglial EVs may use axonal projections to move between connected neurons, limitation of current technologies does not allow monitoring microglial EV dynamics in a complex structure such as the mouse brain.

Importantly, analysis of microglial EVs is extending from rodents to humans: novel protocols have been developed to isolate and study EVs released from iPSC-derived microglia or present in the human brain tissue and body fluids, where myeloid EVs are emerging as useful tools to predict disease evolution and as possible biomarkers for cognitive dysfunction in MS. These new methods have been tailored for small EVs, detectable only by EM 10 years ago and by a variety of methods today, but will hopefully be adapted for large vesicles as well, often less studied but functionally not less relevant.

Despite important advancements have been made in our understanding of the role played by EVs in microglia-brain cell communication and their implication in neurological diseases, still we cannot selectively inhibit the production of distinct EV subtypes (e.g., large EVs vs. small EVs) by either pharmacological or genetic tools nor can we monitor the dynamics of EV interaction with target cells in the mouse brain. The generation of a mouse model, in which large/small EV production from microglia can be inducibly impaired is necessary to validate the contribution of distinct EV populations to neurological diseases and more powerful imaging techniques are required to elucidate the intercellular trafficking of microglial EVs, which may pave the way to novel strategies halting or slowing down the progression of neurodegenerative diseases.

## Author contributions

MG, MF, SR, and CV: conceptualization, writing – original draft preparation, review and editing. MG and SR: figures. All authors read and agreed to the published version of the manuscript.

## Abbreviations

ALS: amyotrophic lateral sclerosis
ATP: adenosine triphosphate
AD: Alzheimer’s disease
CNS: central nervous system
Aβ: beta-amyloid
CSF: cerebro spinal fluid
DRG: dorsal root ganglion
EVs: extracellular vesicles
FACS: fluorescence activated cell sorting
GABA: *γ*-aminobutyric acid
iPSCs: induced Pluripotent Stem cells
INF-*γ*: interferon-*γ*
IL-1β: interleukin 1 β
IL-4: interleukin 4
IB4: isolectin B4
MGnd: neurodegenerative microglia
DAM: disease-associated microglia
LPS: lipopolysaccharide
LTD: long-term depression
LTP: long-term potentiation
MACS: magnetic-activated cell sorting
MCAO: middle cerebral artery occlusion
mEPSCs: miniature excitatory post-synaptic currents
MS: Multiple Sclerosis
PD: Parkinson’s disease
SCI: spinal cord injury
TNF: tumor necrosis factor
TBI: traumatic brain injury
TREM2: triggering receptor expressed on myeloid cells 2.
